# Regional inequalities in under-5 mortality in Nigeria: a population-based analysis of individual- and community-level determinants

**DOI:** 10.1186/1478-7954-9-6

**Published:** 2011-03-09

**Authors:** Diddy Antai

**Affiliations:** 1 Division of Global Health & Inequalities, The Angels Trust - Nigeria, Abuja, Nigeria; 2 Department of Public Health, Division of Social Medicine, Karolinska Institute, Stockholm, Sweden

## Abstract

**Background:**

Regions with geographically diverse ecology and socioeconomic circumstances may have different disease exposures and child health outcomes. This study assessed variations in the risks of death in children under age 5 across regions of Nigeria and determined characteristics at the individual and community levels that explain possible variations among regions.

**Methods:**

Multilevel Cox proportional hazards analysis was performed using a nationally representative sample of 6,029 children from 2,735 mothers aged 15-49 years and nested within 365 communities from the 2003 Nigeria Demographic and Health Survey. Hazard ratios (HR) with 95% confidence intervals (CI) were used to express measures of association among the characteristics. Variance partition coefficients and Wald statistic were used to express measures of variation.

**Results:**

Patterns of under-5 mortality cluster within families and communities. The risks of under-5 deaths were significantly higher for children of mothers residing in the South South (Niger Delta) region (HR: 1.30; 95% CI: 1.76-2.20) and children of mothers residing in communities with a low proportion of mothers attending prenatal care by a doctor (HR: 1.36; 95% CI: 1.15-1.86). In addition, the cross-level interaction between mothers' education and community prenatal care by a doctor was associated with a more than 40% higher risk of dying (HR: 1.41; 95% CI: 1.21-1.78).

**Conclusion:**

The findings suggest the need to differentially focus on community-level interventions aimed at increasing maternal and child health care utilization and improving the socioeconomic position of mothers, especially in disadvantaged regions such as the South South (Niger Delta) region. Further studies on community-levels determinants of under-5 mortality are needed.

## Introduction

The reduction of regional and socioeconomic inequalities in mortality within countries is a major objective of national governments and international organizations [1-3]. To achieve this goal, determinants of high mortality among disadvantaged people, communities, and regions need to be identified. The physical, ecological, and political structure and impoverished socioeconomic milieu in several countries in sub-Saharan Africa account for geographic variations in childhood mortality [4-6]. One such environment is the regional environment. Poor or polluted environments tend to expose children to disease-causing agents, predisposing them to high mortality risks [7]. In Nigeria, marked regional disparities in mortality of children under age 5 have been reported, with higher rates observed in the Northern regions than in the Southern regions [8-10]. However, these were survey reports rather than empirical studies, and the role of explanatory factors was not investigated. Regional disparities in health-seeking behavior have been reported regarding child immunizations [11], maternal and child health care utilization [12], differences in the socioeconomic composition [13], communicable diseases [13], childhood nutrition, and malnutrition [14]. Other studies have reported that the Northern regions have higher proportions of home delivery and complications during childbirth [15], younger age of first marriage, younger age at birth of first child, and lower knowledge and use of contraception compared to the Southern regions [16].

Regional disparities in these parameters are associated with factors at the community level that distinguish these regions from each other. The availability of services and social amenities in communities, or the lack thereof, may positively or negatively influence the health of the residents of communities. Some of these factors include differences in community-level development, population density, prevalence of poverty, and availability of maternal and child health care services. These are often interrelated aspects of the regional environment that are important for child health and well-being, and may also be relevant in exacerbating or mitigating inequities in resources and population health outcomes across regions [7]. Regions may either exert more independence and autonomy within nations (in which case they become even more salient for child health), or may exhibit greater centralization (in which case regional factors may not be as significant) [7]. A classic example of attempts to attain the former is the South South (Niger Delta) region in Nigeria [17]. This study contributes to a better understanding of how regional disparities in social services and health-seeking behavior influence inequalities in child survival. The aims of this study are two-fold: 1) to determine whether there is significant variation in the risks of under-5 deaths across regions of Nigeria; and 2) to determine the individual- and community-level factors that explain disparities in the risks of under-5 deaths among regions.

## Data and Methods

Cross-sectional data from the 2003 Nigeria Demographic and Health Survey (DHS) were used in this study. This is a nationally representative sample collected by face-to-face interviews from 3,725 women aged 15 to 49 years with a stratified two-stage cluster sampling procedure. An extensive report of the survey can be found elsewhere [18]. Birth history data, such as month and year of birth, survivorship status, and current age or age at death if the child had died were collected for each birth. This sample contained a total of 6,029 live-born children, limited to births in the last five years before the survey to ensure that the household variables investigated provided a close enough or accurate picture of the current living conditions of the children within period they were exposed to increased risks of death.

### Ethical considerations

This study is based on analysis of secondary DHS data with all respondent identifiers removed. The survey was approved by the National Ethics Committee in the Federal Ministry of Health of Nigeria and the Ethics Committee of the Opinion Research Corporation Macro International, Inc. (ORC Macro Inc., Calverton, MD, USA).

## Measurements

### Outcome variable

The outcome variable was the risk of under-5 death (0-59 months), defined as a child dying between birth and the 5th birthday. Under-5 mortality was estimated for the five years preceding the survey. All children between 0 and 59 months of age were included in the estimation and exposure time, and cases were observed during this time frame, with all living children 59 months or younger being considered as exposures, contributing person-time, and all deaths among children 59 months or younger regarded as cases. Children born during the time frame (at birth) or before the time frame (at any age until 59 months) could enter this time frame. Children who stayed alive after 59 months of age within this time frame were removed from the sample after 59 months of age.

### Exposure variables

Region of residence of the mother was the main exposure variable, categorized as five sets of dummy variables: a) North Central, b) North East, c) North West, d) South East, and e) South South. The regions were comprised of the following states: North Central region (Benue, Kogi, Kwara, Nasarawa, Niger, Plateau states, and Federal Capital Territory, Abuja); North East region (Adamawa, Bauchi, Borno, Gombe, Taraba, and Yobe states); North West region (Jigawa, Kaduna, Kano, Katsina, Kebbi, Sokoto, and Zamfara states); South East region (Abia, Anambra, Ebonyi, Enugu, and Imo states); South South region (Akwa Ibom, Bayelsa, Cross River, Delta, Edo, and Rivers states); and South West region (Ekiti, Lagos, Ogun, Ondo, Osun, and Oyo states).

Explanatory factors: Eight additional individual-level factors of interest were examined: *i) *birth order, consisting of two dummy variables: *a*) first births and *b*) 5^th ^or higher birth order; *ii) *sex of the child, female; *iii) *mother's age, consisting of two dummy variables: *a) *15-23 years and *b) *34 years or older; *iv*) mother's age at birth of first child, 18 years or younger; *v*) marital status, consisting of two dummy variables: *a) *single and *b) *divorced; *vi*) religious affiliation, made up of two dummy variables: *a) *Muslim and *b)t*raditional; *vii*) mother's education, consisting of two dummy variables: *a) *no education and *b) *primary education; and *viii*) wealth index, consisting of two dummy variables: *a) *poorer wealth quintile and *b) *richer wealth quintile. Wealth index was applied in this analysis as a composite index and an indicator of the socioeconomic status of households because the DHS does not generally collect information on household income or wealth. It is consistent with expenditure and income measures in low- and middle-income countries. It assigns weights or factor scores generated by principal component analysis to information on household assets collected from censuses and surveys. These indicators include those relating to household ownership of durable assets and household environmental conditions. Principal components analysis allows each asset owned to be given a score, and the factor loading scores are used to create linear composites of each household socioeconomic status variable. The scores are then summed and divided into quintiles (poor, middle, and rich) to represent different levels of wealth.

Cross-level interactions between individual- and community-level measures of socioeconomic position provide the opportunity to explore whether community-level effects are different for children of mothers in low socioeconomic position. Interaction effects were assessed as *a) *cross-level interaction between mother's age at birth of first child and community prenatal care by doctor; and *b) *cross-level interaction between mother's education and community prenatal care by doctor.

Three community-level factors were assessed: *i) *level of mother's education in the community, defined as the percentage of mothers with secondary or higher education in the primary sampling unit (PSU), consisting of two subsets of dummy variables: *a*) low and *b*) high. This variable was selected because higher levels of maternal education are associated with better child health outcomes like childhood mortality and child immunization rates [19,20]. Thus, the proportion of mothers with secondary or higher education is a predictor of child survival.

The second community-level factor assessed was: *ii) *community hospital delivery, defined as the percentage of mothers who delivered their children in the hospital in the PSU, consisting of two subsets of dummy variables: *a*) low and *b*) high. And the third community-level factor assessed was: *iii) *community prenatal care by doctor, defined as the percentage of mothers who had prenatal care provided by a doctor, consisting of the dummy variable low. Prenatal care directly increases the chances that mothers would access subsequent health care services for their children, such as delivery in a health institution as well as mother and child immunization [21,22]. Hospital delivery is also one of the most important preventive measures against poor maternal and child health outcomes and an important determinant of full immunization [23,24]. Hence, the proportion of mothers that received prenatal care and the proportion that delivered in a hospital setting are both salient predictors of child survival.

The contextual variables were at the level of the PSU (*n *= 365). Primary sampling units are small, administratively defined areas designed to be fairly homogeneous units in relation to population-level socio-demographic characteristics, economic status, and living conditions. They are used as proxies for "neighborhoods" or "communities" [25,26] and contain one or more enumeration areas, which are the smallest geographic units for which census data are available in Nigeria. Each cluster consisted of a minimum of 50 households, with a contiguous enumeration area added when a cluster had fewer than 50 households [18].

### Statistical analysis

The distribution of the individual- and community-level characteristics in the sample was assessed separately by region of residence in order to assess the unadjusted effect of these characteristics on region of residence. Data were analyzed using Multilevel Cox proportional hazards analysis [27], which models censored time-until-event data as a dependent variable where one can assume that the covariates have a multiplying effect on hazard rates and warrants recoding characteristics in dummy variables. The associations among under-5 mortality and individual- and community-level characteristics were assessed separately (in order to show how regional variation is built up from variation on various levels) as well as successively. Measures of association (fixed effects) are expressed as hazard ratios (HR), 95% confidence intervals (95% CIs) and *p*-value. Measures of variation (random effects) are expressed as intraclass correlation (ICC), which is a measure of the relatedness of clustered data. Generalized linear and latent mixed models (gllamm) were used to perform the three-level multilevel analysis using Stata version 10.0 [28].

Four models were fitted in the analysis containing individual- and community-level characteristics. Model 0 (empty model) contained no explanatory variable since its role was to decompose the total variance into its individual- and community-level components, and to identify a possible contextual phenomenon that can be quantified by clustering of under-5 mortality within neighborhoods [29]. Model 1 contained region of residence as the only explanatory variable to assess the gross effects of region of residence before netting out the effects of other variables, and Model 2 added sex of the child and birth order. Model 3 included the mother-level variables (mother's age, mother's age at birth of first child, marital status, religious affiliation, mother's education, wealth index, and cross-level interactions between community prenatal care by doctor and mother's age at birth of first child as well as mother's education). Finally, Model 4 added community-level variables (community mother's education, community hospital delivery, and community prenatal care by a doctor). The simultaneous inclusion of both individual- and neighborhood-level predictors in the multilevel Cox regression model permits: *i) *the examination of neighborhood or area effects after individual-level confounders have been controlled for; *ii) *the examination of individual-level characteristics as modifiers of the area effect (and vice versa); and *iii) *the simultaneous examination of within- and between neighborhood variability in outcomes, and of the extent to which between-neighborhood variation is explained by individual- and neighborhood-level characteristics [30,31].

## Results

### Characteristics of children and women by region of residence

Examination of individual-level characteristics across regions of residence showed that children in the Northern regions constituted most of the study sample. Mothers who were Christians and had secondary or higher education were most likely from the Southern regions, while mothers from the Northern regions were mostly Muslims, in the middle and poor wealth quintiles, and were 18 years or younger at the birth of their first child (Table 1). The assessment of community-level characteristics across regions of residence in the study sample indicated that children in the Northern regions constituted most of the study sample. Most of the mothers in the North East and North West regions lived in communities with a low proportion of mothers who attended prenatal care by a doctor, while most of the mothers in the North Central and North East regions lived in communities at the median community level in regard to the proportion of mothers who had hospital delivery, as well as the proportion of mothers with secondary or higher education. In contrast, most of the mothers in the Southern regions lived in communities with a high proportion of mothers who attended prenatal care by a doctor, and within the South East and South West regions, in communities with a high proportion of mothers who had hospital deliveries. Mothers in the North Central and North East regions who lived in communities with the proportion of secondary or higher education at the median level for the community made up most in the sample (Table 2).

**Table 1:** Distribution of individual-level characteristics by region of residence

Characteristics	Region of residence						Total N (%)
		
	North Central N (%)	North East N (%)	North West N (%)	South East N (%)	South South N (%)	South West N (%)	
*Region of residence*	1,015 (17)	1,487 (25)	1,821 (30)	524 (9)	560 (9)	622 (10)	6029 (100)
*Birth order*							***
First birth	210 (21)	240 (16)	349 (19)	99 (19)	136 (24)	166 (27)	1,200 (20)
2 - 4 birth order	473 (46)	603 (41)	759 (42)	255 (49)	244 (44)	317 (51)	2,621 (43)
≥ 5 birth order	332 (33)	644 (43)	713 (39)	200 (38)	180 (32)	139 (22)	2,208 (37)
*Sex of the child*							***
Female	492 (49)	756 (51)	915 (50)	283 (54)	281 (50)	335 (54)	3,062 (51)
Male	523 (51)	731 (49)	906 (50)	241 (46)	279 (50)	287 (46)	2,967 (49)
*Mother's age*							***
≤ 23 years	220 (22)	396 (27)	529 (29)	62 (12)	118 (21)	86 (14)	1,411 (23)
24 - 33 years	556 (55)	716 (48)	877 (48)	269 (51)	290 (52)	362 (58)	3,070 (51)
≥ 34 years	239 (23)	375 (25)	415 (23)	193 (37)	152 (27)	174 (28)	1,548 (26)
*Mothers' age at birth of first child*							***
18 years or less	516 (51)	998 (67)	1258 (69)	147 (28)	280 (50)	138 (22)	3,337 (55)
19 years or more	499 (49)	489 (33)	563 (31)	377 (72)	280 (50)	484 (78)	2,692 (45)
*Marital status*							***
Single	25 (2)	7 (0)	3 (0)	13 (3)	49 (9)	12 (2)	109 (2)
Married	956 (94)	1,421 (96)	1,775 (97)	477 (91)	481 (86)	598 (96)	5708 (95)
Divorced	34 (4)	59 (4)	43 (3)	34 (6)	30 (5)	12 (2)	212 (3)
*Religious affiliation*							***
Christian	592 (58)	215 (15)	111 (6)	482 (92)	540 (97)	367 (59)	2307 (38)
Muslim	392 (39)	1257 (84)	1701 (93)	0 (0)	7 (1)	241 (39)	3598 (60)
Traditional	31 (3)	15 (1)	9 (1)	42 (8)	13 (2)	14 (2)	124 (2)
*Mothers' education*							***
No education	416 (41)	1,032 (69)	1,338 (73)	90 (17)	52 (9)	105 (17)	3033 (50)
Primary	355 (35)	263 (18)	248 (14)	210 (40)	195 (35)	202 (32)	1473 (25)
Secondary or higher	244 (24)	192 (13)	235 (13)	224 (43)	313 (56)	315 (51)	1523 (25)
*Wealth Index*							***
Poor	425 (42)	866 (58)	876 (48)	214 (41)	180 (32)	166 (27)	2,727 (45)
Middle	440 (43)	524 (36)	762 (42)	203 (39)	238 (43)	147 (24)	2,332 (39)
Rich	150 (15)	79 (6)	183 (10)	107 (20)	142 (25)	309 (49)	970 (16)

*p < .05; **p < .01; ***p < .001

**Table 2:** Distribution of community-level characteristics by region of residence

Characteristics	North Central N (%)	North East N (%)	North West N (%)	South East N (%)	South South N (%)	South West N (%)	Total N
*Community mother's education*							***
Low	93 (9)	485 (33)	797 (44)	70 (13)	53 (9)	116 (19)	1,614
Middle	510 (50)	821 (55)	841 (46)	205 (39)	263 (47)	252 (40)	2,892
High	412 (41)	181 (12)	183 (10)	259 (48)	244 (44)	254 (41)	1,523
*Community hospital delivery*							***
Low	88 (9)	543 (36)	866 (48)	8 (1)	2 (0)	8 (1)	1,515
Middle	640 (63)	869 (59)	891 (49)	134 (26)	317 (57)	154 (25)	3,005
High	287 (28)	75 (5)	64 (3)	382 (73)	241 (43)	460 (74)	1.509
*Community prenatal care by doctor*							***
Low	511 (50)	1,349 (91)	1,774 (97)	78 (15)	61 (11)	81 (13)	3,854
High	504 (50)	138 (9)	47 (3)	446 (85)	499 (89)	541 (87)	2,175

*p < .05; **p < .01; ***p < .001

### Risk factors of under-5 mortality

The risk factors for under-5 five mortality by region of residence were controlled separately for individual-level factors, and findings showed that the risks of under-5 deaths were almost twofold higher for children of mothers residing in the North East (HR: 1.72; 95% CI: 1.14 - 2.59); North West (HR: 1.71; 95% CI: 1.13 - 2.57); and South South (HR: 1.91; 95% CI: 1.21 - 3.02) regions compared with children of mothers in the South West region. The risks of dying were higher for children of ≥ 5 birth order (HR: 1.58; 95% CI: 1.23 - 2.02); children of mothers with no education (HR: 1.63; 95% CI: 1.21 - 2.19) or primary education (HR: 1.60; 95% CI: 1.21 - 2.13); and in the poor wealth quintile (HR: 1.28; 95% CI: 1.02 - 1.59). In contrast, the risks of dying were lower for children of mothers in the middle wealth quintile (HR: 0.70; 95% CI: 0.54 - 0.92) (Table 3).

**Table 3:** Odds ratios and 95% confidence intervals for individual-level risk factors for under-5 mortality and region of residence

Characteristics	OR (95% CI)
*Region of residence*	
North Central	1.25 (0.83 - 1.88)
North East	1.72 (1.14 - 2.59)
North West	1.71 (1.13 - 2.57)
South East	1.14 (0.71 - 1.84)
South South	1.91 (1.21 - 3.02)
South West	1
*Sex of child*	
Female	1.08 (0.92 - 1.26)
Male	1
*Birth order*	
First birth (order 1)	1.27 (1.00 - 1.61)
2 - 4 birth order	1
≥ 5 birth order	1.58 (1.23 - 2.02)
*Mother's age*	
≤ 23 years	1.03 (0.82 - 1.31)
24 - 28 years	1
≥ 34 years	0.96 (0.74 - 1.26)
*Mother's age at birth of first child*	
≤ 18 years	1.11 (0.91 - 1.36)
≥ 19 years	1
*Marital status*	
Single	0.61 (0.29 - 1.27)
Married	1
Divorced	1.40 (0.93 - 2.11)
*Religious affiliation*	
Christian	1
Muslim	1.03 (0.77 - 1.37)
Traditional	1.67 (0.99 - 2.83)
*Mothers' education*	
No education	1.63 (1.21 - 2.19)
Primary education	1.60 (1.21 - 2.13)
Secondary or higher education	1
*Wealth index*	
Poor	1.28 (1.02 - 1.59)
Middle	0.70 (0.54 - 0.92)
Rich	1

The risk factors for under-5 mortality by region of residence also were controlled separately for community-level factors. Children of mothers resident in the South South region had a 15% higher risk of under-5 death (HR: 1.15; 95% CI: 1.06 - 1.90) compared to children of mothers in the South West region. In addition, the risks of dying were higher for children resident in communities with a low proportion of mothers who attended prenatal care by a doctor (HR: 1.68; 95% CI: 1.46 - 1.98) and who had hospital delivery (HR: 1.34; 95% CI: 1.04 - 1.72), while the risks of dying were lower for children resident in communities with a high proportion of hospital delivery (HR: 0.66; 95% CI: 0.44 - 0.98) (Table 4).

**Table 4:** Association between community-level risk factors, under-5 mortality, and region of residence

Characteristics	OR (95% CI)
*Region of residence*	
North Central	1.12 (0.70 - 1.79)
North East	1.29 (0.79 - 2.12)
North West	1.15 (0.70 - 1.90)
South East	0.72 (0.39 - 1.33)
South South	1.15 (1.06 - 1.90)
South West	1
*Community mother's education*	
Low	1.10 (0.86 - 1.39)
Middle	1
High	1.13 (0.85 - 1.52)
*Community prenatal care by doctor*	
Low	1.68 (1.46 - 1.98)
High	1
*Community hospital delivery*	
Low	1.34 (1.04 - 1.72)
Middle	1
High	0.66 (0.44 - 0.98)

The risk factors for under-5 mortality by region of residence were also controlled simultaneously for individual- and community-level factors. Region of residence was included as the only explanatory variable in Model 1 to assess the independent influence of region of residence on the risks of under-5 deaths, and was significantly associated with the risks of under-5 deaths, with about twofold higher risks for children of mothers resident in the North East (HR: 2.41; 95% CI: 1.63 - 3.57), North West (HR: 2.28; 95% CI: 1.56 - 3.34), and South South (HR: 1.85; 95% CI: 1.17 - 2.91) compared with children of mothers resident in the South West region. The risks of under-5 deaths were attenuated with the inclusion of child, mother, and community characteristics; in the final model (Model 4), children of mothers resident in the South South region had 30% higher risks of dying (HR: 1.30; 95% CI: 1.76 - 2.20) compared with children of mothers resident in the South West region (Table 5).

**Table 5:** Odds ratios and 95% confidence intervals for individual- and community-level risk factors for under-5 mortality and region of residence

Variables	Model 0	Model 1	Model 2	Model 3	Model 4
**Fixed part**		**HR (95% CI)**	**HR (95% CI)**	**HR (95% CI)**	**HR (95% CI)**

*Region of residence*					
North Central		1.51 (0.99 - 2.30)	1.50 (0.99 - 2.29)	1.31 (0.83 - 2.07)	1.27 (0.79 - 2.03)
North East		2.41 (1.63 - 3.57)	2.35 (1.59 - 3.49)	1.49 (0.94 - 2.26)	1.38 (0.83 - 2.29)
North West		2.28 (1.56 - 3.34)	2.23 (1.52 - 3.27)	1.41 (0.89 - 2.26)	1.29 (0.77 - 2.17)
South East		1.41 (0.88 - 2.26)	1.37 (0.85 - 2.21)	0.65 (0.35 - 1.23)	0.68 (0.36 - 1.28)
South South		1.85 (1.17 - 2.91)	1.82 (1.15 - 2.87)	1.36 (1.81 - 2.30)	1.30 (1.76 - 2.20)
South West		1	1	1	1
*Sex of child*					
Female			1.09 (0.93 - 1.27)	1.04 (0.87 - 1.24)	1.04 (0.87 - 1.24)
Male			1	1	1
*Birth order*					
First birth (order 1)			1.40 (1.16 - 1.68)	1.2 (0.99 - 1.68)	1.28 (0.98 - 1.68)
2 - 4 birth order			1	1	1
≥ 5 birth order			1.44 (1.16 - 1.80)	1.64 (1.24 - 2.18)	1.64 (1.24 - 2.17)
*Mother's age*					
≤ 23 years				1.01 (0.77 - 1.31)	1.02 (0.78 - 1.32)
24 - 28 years				1	1
≥ 34 years				0.84 (0.62 - 1.13)	0.84 (0.62 - 1.14)
*Mother's age at birth of first child*					
≤ 18 years				1.12 (0.88 - 1.42)	1.13 (0.88 - 1.44)
≥ 19 years				1	1
*Marital status*					
Single				0.72 (0.29 - 1.78)	0.73 (0.29 - 1.78)
Married				1	1
Divorced				1.75 (1.08 - 2.82)	1.76 (1.09 - 2.84)
*Religion*					
Muslim				1.09 (0.78 - 1.51)	1.08 (0.77 - 1.50)
Traditional				1.62 (0.78 - 3.36)	1.60 (0.77 - 3.34)
Christian				1	1
*Mothers' education*					
No education				2.24 (1.51 - 3.32)	2.17 (1.44 - 3.26)
Primary education				1.92 (1.35 - 2.74)	1.90 (1.32 - 2.75)
Secondary or higher education				1	1
*Wealth index*					
Poor				1.27 (0.98 - 1.63)	1.23 (0.95 - 1.59)
Middle				1	1
Rich				0.85 (0.62 - 1.17)	0.89 (0.64 - 1.22)
*Cross-level interaction (mother's age at birth of first child & community prenatal care by doctor*				0.94 (0.63 - 1.40)	1.15 (0.58 - 1.51)
*Cross-level interaction (mother's education & community prenatal care by doctor*				1.08 (0.25 - 1.76)	1.41 (1.21 - 1-78)
*Community mother's education*					
Low					1.05 (0.83 - 1.34)
Middle					1
High					1.02 (0.75 - 1.39)
*Community prenatal care by doctor*					
Low					1.36 (1.15 - 1.86)
High					1
*Community hospital delivery*					
Low					1.12 (0.87 - 1.45)
Middle					1
High					0.79 (0.52 - 1.22)
**Random part**	Empty	Region of residence	Child-level	Mother-level	Community-level
*ICC Mothers*	8.2	7.2	3.5	3.3	3.3
Variance (SE)	0.316 (0.137)	0.271 (0.131)*	0.127 (0.125)	0.116 (0.124)	0.120 (0.122)
*ICC Communities*	6.6	5.3	5.4	3.3	3.1
Variance (SE)	0.253 (0.074)	0.200 (0.067)**	0.196 (0.065)**	0.118 (0.058)*	0.109 (0.055)*
Log likelihood	-2407.81	-2392.61	-2383.53	-1768.31	-1763.13

*p < .05; **p < .01; ***p < .001

Furthermore, children of ≥ 5 birth order and of divorced mothers had 24% (HR: 1.24; 95% CI 1.24 - 2.17) and 76% (HR: 1.76; 95% CI 1.09 - 2.84) higher risks of under-5 deaths compared with children of 2^nd^- 4^th ^birth order and married mothers, respectively. The risks were more than twofold higher for children of mothers with no education (HR: 2.17; 95% CI 1.44 - 3.26), and almost twofold higher for children of mothers with primary education (HR: 1.90; 95% CI 1.32 - 2.75) compared with children of mothers with secondary or higher education. The cross-level interaction between mothers' education and community prenatal care by a doctor was associated with a 41% higher risk of dying (HR: 1.41; 95% CI 1.21 - 1-78). A low proportion of mothers attending prenatal care by a doctor in the community was associated with 36% (HR: 1.36; 95% CI 1.15 - 1.86) higher risks of under-5 deaths compared with high proportions.

## Discussion

This study showed that under-5 mortality was significantly associated with region of residence, with higher risks of under-5 deaths for children of mothers resident in the South South region after adjusting only for individual-level risk factors and only community-level risk factors, as well as simultaneously adjusting for individual- and community-level risk factors. The risk of under-5 deaths was higher in the North East and North West regions after adjusting for child-level variables; however, these differences were reduced and became insignificant once mother-level and community-level controls were added. These elevated risks in the North East and North West regions were explained in part by differences in mothers' education, which corroborates the finding that mothers in the northern regions within this study have a higher proportion of no education or primary education. Findings in this study indicate that demographic factors such as birth order, socioeconomic factors such as marital status, mothers' education, and community-level factors such as living in communities with a low proportion of mothers that received prenatal care by a doctor are the main predictors of regional under-5 mortality. This may be associated with spatial inequality in social development in the community within regions, which may also be associated with population density, differential levels of regional development, political and religious situations, as well as varying economic resources [32]. These factors reflect the situation in the South South region of Nigeria, which is reported to suffer from deficient social infrastructure and services (schools, roads, electricity, and health services), high unemployment, social deprivation, and endemic conflict, in spite of the region accounting for more than 90% of Nigeria's proven gas and oil reserves and the nation's wealth [33,34]. Geographically, the region is characterized by extensive mangrove forests and extensive networks of lagoons and swamps affected by environmental degradation from crude oil spillage and pollution. These conditions may be related with the increased risks of under-5 deaths for children in this region.

In addition, cross-level interaction between mother's education and community prenatal care by a doctor (i.e., mothers who had no education and lived in communities with low community prenatal care) was associated with a more than twofold increase in risks of under-5 deaths. Although the specific mechanisms underlying the increased mortality are unknown, plausible explanations may include the fact that mothers with little or no education residing in communities deprived of prenatal care by a doctor are more vulnerable because they are generally poorer and lack the economic means for essential goods and services (health care, medications, and transportation) compared to mothers with higher education and access to prenatal care.

The risks of under-5 deaths were higher for ≥ 5 birth order. The influence of birth order on various types of child outcomes is largely dependent on the social and cultural context and may be associated with social disadvantages within families and communities [35], given that large sibships may be a marker for low socioeconomic status [36] and erosion of nutritional resources [37,38]. However, the influence of other unmeasured factors such as utilization of family planning services cannot be ruled out. This study also found that lower socioeconomic position (divorce, no education, primary-only education) was associated with increased risks of under-5 deaths, which is in agreement with findings from previous studies indicating that higher socioeconomic position of individuals and populations strongly influences health-seeking behavior and is associated with better health [39,40].

Furthermore, living in communities with a low percentage of mothers who received prenatal care by a doctor was associated with higher risks of under-5 deaths. This could be explained by lower access to prenatal care directly increasing the chances that mothers in the community would not utilize health care services, such as institutional delivery and immunization for their child [41,42]. Timely access to prenatal care by a doctor is an important preventive measure against maternal and child health outcomes such difficult or obstructed labor, postnatal bleeding, and child deaths [43,44]. Community prenatal care by a doctor is also an indication of the quality of care received by the mother and infant during childbirth. This association at the community level is also a reflection of socioeconomic position at the individual level because individual socioeconomic position strongly influences health-seeking behavior by enhancing mothers' perceptions of disease etiology and treatment patterns, resulting in improved health and welfare of their children. Higher socioeconomic position empowers mothers and enhances their decision-making power. Community-level variation remained significant after controlling for individual- and community-level variables, indicating that differences among communities still remain unexplained and need further exploration regarding the community-level determinants of under-5 mortality. This finding is also validated by the result of the cross-level interaction.

The intraclass correlation across communities in all the models and across mothers in Model 1 was significantly different from zero, implying that there are significant differences in the risks of under-5 deaths between communities in all the models and between mothers in Model 1 (with region of residence as the only explanatory variable).

The geopolitical regions of low- and middle-income countries such as Nigeria are an important sphere of influence on child survival. As such, findings show the relevance of adopting a more spatially disaggregated, community-level approach to regional policies, given that regions are usually made up of people and communities sharing similar geographical, political, socioeconomic, and cultural characteristics that either promote or inhibit health-seeking behavior and access to health care facilities.

Several limitations need to be considered when interpreting findings in this study. First, administratively defined boundaries were used as a proxy for neighborhoods or communities in this study. There is an inherent risk of nondifferentially misclassifying individuals into inappropriate administrative boundaries, which may generate information biases and reduce the validity of the analysis. Second, data on household income or expenditure, which are the indicators commonly used to measure wealth, are not routinely collected in DHS surveys. The assets-based wealth index used in this study is only a proxy indicator for individual/household economic status and may not always produce results similar to those obtained from direct assessments of income and expenditure where such data are available or can be reliably collected [45,46]. The strengths of this study are also worth mentioning and include: *i) *DHS surveys are nationally representative and enable the generalization of the results across the country; *ii) *the DHS variables are defined similarly across countries, and results are therefore comparable across countries [47,48]; and *iii) *using administrative boundaries permits the comparability of any set of DHS data on the same geographical frame, or of presenting complex data in a simple way, provided there is a good conceptual framework of the studied territory.

## Conflict of interests

The authors declare that they have no competing interests.

## Authors' contributions

The conception, data analyses, and interpretation of results in this manuscript were done by DA.
